# Responses to a GP survey: current controversies in diet and cardiovascular disease

**DOI:** 10.1186/s12875-018-0840-4

**Published:** 2018-08-31

**Authors:** Bruce A. Griffin, John A. A. Nichols

**Affiliations:** 1 Department of Nutritional Science, University of Surrey, Guildford, UK; 2 Department of Clinical Medicine and Ageing, University of Surrey, 60 Manor Way, Guildford, Surrey GU2 7RR UK

**Keywords:** Cardiovascular diseases, Primary health care, Nutrition science, Dietetics, Medical education

## Abstract

**Background:**

When advising patients on diet and health, the general practitioner (GP) makes judgements based on the evidence available. Since current evidence on diet and cardiovascular disease is conflicted and confusing, we surveyed the current consensus amongst GPs. The aim of this study was to determine the views of GPs on dietary saturated fat, carbohydrates and long chain omega-3 fatty acids in the management of cardiovascular disease.

**Method:**

An online questionnaire inviting participants to comment on seven contentious statements on diet and cardiovascular disease. Questionnaire circulated to the 1800 members of South West Thames Faculty of the Royal College of General Practitioners (RCGP). Participants were invited to tick “Agree”, “Disagree” or “Not sure” and were encouraged to add comments for each question. The results were analysed with a combination of statistical analysis and thematic analysis of comments.

**Results:**

There were 89 responses. Most GPs seem well aware that drug treatment alone is inadequate and that dietary advice is important. However, there was considerable disagreement about the roles of saturated fats and carbohydrates in cardiovascular disease and “Not sure” responses ranged from 12 to 40.7%. The 40.7% related to a statement on long chain omega-3 fatty acids. Analysis of comments revealed more opinions including an awareness of the need to warn patients about *trans*-fatty acids.

**Conclusions:**

Although the GP response rate was poor, responders do seem to see dietary advice as part of their role but do not consider themselves as experts. Education in this area should have a higher priority.

**Electronic supplementary material:**

The online version of this article (10.1186/s12875-018-0840-4) contains supplementary material, which is available to authorized users.

## How this fits

General practitioners need to give advice on nutrition, diet and health, especially cardiovascular health concerns. There has been concern that medical school gives little if any time to these issues. This survey shows that there is a good deal of confusion in this area and our data suggest that most knowledge is gained through experience working in primary care.

## Background

The average general practitioner (GP) in the United Kingdom (UK) will have received very little education at medical school on the role of nutrition in health and disease [1–3], but should be well versed in Pharmacology as a prescribing doctor. In a recent survey [2], only 33% of medical school academics felt that they were providing adequate teaching on nutrition and health. Therefore, GPs may feel more confident in providing advice on drug therapy rather than diet for the prevention of cardiovascular disease (CVD). However, an experienced GP may have acquired information about diet and lifestyle through their professional experience. Current controversies over the relevance of free sugars (as opposed to endogenous sugars in fruit (fructose) and milk (lactose)) and fats to the aetiology of CVD [4–6] has caused confusion amongst the general public [7, 8] and we wondered what the consensus was amongst GPs. Also, since 2013, the National Institute for Health and Care Excellence (NICE) clinical guidelines have de-emphasised the role of long chain omega-3 fatty acids in the management of CVD and advise against routine prescribing of omega-3 acid ethyl esters (Omacor) [9]. However, NICE continue to recommend an increase in dietary fish as part of a Mediterranean diet [9]. There is a substantial body of evidence for benefits to both primary and secondary cardiovascular health from long chain omega-3 [10–13], and an equally impressive body of evidence that has influenced NICE to be cautious [14–16]. A 2017 study lasting 19 years has shown a lower CVD death rate (hazard ratio 0.73) in subjects taking a long term regular daily dose of a long chain omega-3 fatty acid supplement [17].

The aim of our study was to assess the views of GPs on a range of issues relating to nutrition and cardiovascular disease by measuring their response to several emotive and contentious questions about diet and health.

## Methods

A survey monkey questionnaire was sent by email to 1800 GPs in the SW Thames section of the RCGP. One follow-up reminder was sent with a link to the questionnaire as recommended by Pealer et al. [18]. This was followed by asking non-responders to complete the same questionnaire at an NHS Clinical Commissioning Group (CCG) meeting for all GPs in the Guildford and Waverly area of Surrey (approximately 6% of SW Thames GPs). The questions were framed as tests of opinion rather than tests of knowledge with tick box options “Agree”, “Disagree” and “Not sure”. Participants were also encouraged to add comments and the comments were analysed using thematic analysis.

Questions:

1. Once you start a patient on a statin, diet isn’t very important.
2. Omega 3 intake is key to recovery from a cardiac event.
3. LDL cholesterol is more important than HDL cholesterol when interpreting a lipid profile.
4. The latest evidence on dietary fat means that we can now advise that it is safe to consume saturated fat-rich foods.
5. Diet and dyslipidaemia are linked to dysfunction of the endothelium.
6. Non-alcoholic fatty liver disease is a major risk factor for cardiovascular disease.
7. Dietary carbohydrates are neutral with respect to cardiovascular disease.

### Statistics

The completed questionnaires were analysed using chi-squared foursome tables to determine differences between internet responders and non-responders and calculate *p* values. IBM SPSS Statistics software was used to analyse gender differences, significance of years in general practice using the student t-test and correlation coefficients to show any correlations between different components of the questionnaire and to calculate *p* values for this data. An analysis of respondents’ comments was made for recurrent themes.

## Results

A total of 89 GPs responded to the initial questionnaire and a further 19 non-responders completed the questionnaire at the CCG meeting. The total sample was 108, which was 6% of the targeted GPs.

There were 74 (68.5%) female GPs and 33 (30.5%) male GPs. Although age was not recorded for participants the number of years in general practice was recorded. The significance of age and experience was reflected in this data (Additional file 1: Appendix 1). The mean number of years in general practice was 13.7 yrs. (range 0.5–42) and for male GPs average was 17.6 yrs. (range 2–42) and for female GPs average was 12 yrs. (range 0.5–33). This data was similar to regional data for age and gender for GPs in South England although there was a slight bias in favour of female GPs responding to the questionnare which failed to reach statistical significance (Additional file 2: Appendix 2).

The combined results are summarised in Table 1. There was no statistically significant difference between internet responders and non-responders. However there was a trend for Question 2 with a greater proportion of non-responders disagreeing with the statement “Omega 3 intake is key to recovery from a cardiac event”: Responders disagreed 30/89 (33.7%) Non responders disagreed 10/19 (52.6%), *p* = 0.076. Responders were twice as likely to respond “Not sure” than non-responders:

**Table 1:** Summary of results from questionnaire

Questions	Agree	Disagree	Not sure
1. Diet is unimportant for patients on statins	3 (2.8%)	105 (97.2%)	0 (0%)
2. Omega-3 relevant to cardiac events	24 (22.2%)	40 (37%)	44 (40.7%)
3. LDL more important than HDL	49 (45.4%)	42 (38.9%)	17 (15.7%)
4. Dietary saturated fat safe	18 (16.7%)	57 (52.8%)	33 (30.6%)
5. Endothelial dysfunction and dyslipidaemia	80 (74.1%)	3 (2.8%)	26 (24.1%)
6. NAFLD major risk factor for CVD	77 (71.3%)	6 (5.6%)	26 (24.1%)
7. Dietary carbohydrates neutral for CVD	11 (10.2%)	84 (77.8%)	13 (12%)

Responders “not sure” 40/89 (44.9%) and non-responders “not sure” 4/19 (21.1%), *p* = 0.054.

There was a trend for female GPs to be more likely to chose “Not sure” responses (Table 2), but this was only statistically significant for Question 4 which suggested that dietary saturated fat is now considered “safe”. 78.8% of male GPs disagreed with this statement, whereas only 41.9% of female GPs disagreed and 35% were “Not sure.”

**Table 2:** Gender trend for response: not sure

Questions	Female (*n* = 74)	Female %	Male (*n* = 33)	Male %
1. Statins more important than diet	0	0%	0	0%
2. Omega-3 relevant to cardiac events	34^b^	45.9%	9^b^	27.3%
3. LDL more important than HDL	11	14.9%	6	18.2%
4. Dietary saturated fat safe	26^a^	35.1%	6^a^	18.2%
5. Endothelial dysfunction & dyslipidaemia	21	28.4%	4	12.1%
6. NAFLD major risk factor for CVD	17	24.2%	8	23.0%
7. Dietary carbohydrates neutral for CVD	11	14.9%	2	6.1%

^a^Significant gender difference at *p* < 0.05

^b^Borderline non-significant gender difference at *p* = 0.07

There was a trend for years in practice as a GP to be a factor in responses to the statements about omega-3 and cardiac events, interpretation of lipid profiles and non-alcoholic fatty liver disease with responses from younger GPs reflecting their lack of experience (Table 3). There was a degree of overlap between questions which did not reach statistical significance: 42.6% disagreed that dietary carbohydrates are neutral for CVD and disagreed that it is now safe to advise saturated fat rich food and 42.6% agreed that diet & dyslipidaemia are linked to endothelial dysfunction and were of the opinion that dietary saturated fat is not safe.

**Table 3:** Years as a GP influence on responses

Question numbers			*p* values
Q3	Omega-3 relevant to cardiac events	“Agree” sub-group had been mean of 5 yrs. longer as a GP than “Disagree” sub-group	0.17
Q2	LDL more important than HDL	“Unsure” sub-group had been mean of 6 yrs. longer as a GP than “Agree” and “Disagree” combined	0.057
Q5	Dyslipidaemia linked to endothelial dysfunction	“Disagree” (*n* = 3) were recent recruits to GP with mean of 5.8 yr. as a GP	0.15
Q6	NAFLD major risk factor for CVD	“Disagree” (*n* = 6) were recent recruits to GP with mean of 6.9 yrs. as a GP	0.1
Questions 1, 4, 7		Years as GP were not significant	–

Although there was an even split for statement 3 on the relative importance of HDL and LDL cholesterol with 15.7% of respondents opting for “not sure” (Table 1), 5.6% added the comment that the HDL/cholesterol ratio is more significant.

Subjects were invited to add comments to all questions and at the end of the questionnaire. There were 37 comments that varied from very brief such as “in moderation” for the question on safety of saturated fats, to longer detailed comments on the role of nutrition science in primary care. Comments of 4 words or less that conveyed little or no meaning, were excluded (5/37). Comments that could be classified thematically (32/37) tended to come from respondents who had been GPs a little longer (Table 4).

**Table 4:** Number of years as a GP Versus type of comment or no comment

Type of comments:	N	Mean years as a GP	Std. Deviation	Std. Error Mean
None or insignificant	76	13.2	9.7196	1.1149
Significant	32	15.1	11.1773	1.9759

χ^2^ test: *p* = 0.038

From a total of 32 such comments, the commonest comment (10/32) was an acknowledgement that a wide range of nutrients are important to cardiovascular health, and that a pharmacological approach alone is inadequate. Comments that are representative examples of each type of response are summarised in Table 5.

**Table 5:** Analysis of participant’s comments

Topic of comments	Number of comments	Questions comments related to	One good representative example of comments from this category
General value of diet in cardiovascular health.	10	Q1 and final comment	Even if it has less effect on lipids than the statin, diet is still important in other areas of cardiovascular health.
Trans fatty acids an important risk factor.	7	Qs 3, 4, 5 and final comment	Excess saturated fat, cholesterol and trans-fats all increase the risk of CVD.
Refined sugar an important risk factor	7	Qs 4, 7 and final comments	Complex carbohydrates have a beneficial effect and refined sugar the opposite.
HDL/cholesterol ratio is the best guide	6	Q3	Both HDL and LDL are equally relevant. The ratio between them is important.
References to diet and metabolic syndrome	5	Qs 6, 7 and final comments	Carbohydrates are negative in that they contribute to the metabolic syndrome and DM
Low GI diet is beneficial	3	Q7	A high GI diet has been shown to be a risk factor for CVD
Sugar can be converted to fat	3	Q7	High intake of refined sugar leads to dyslipidaemia with sugar converted to fat
GPs need more nutrition knowledge	3	Final comments	I wish we doctors had more access to education related to nutrition
Advise patients to eat more plant foods	2	Q4	The main source of fats should be plant rather than animal derived.
High homocysteine is a risk factor for CVD	1	Final comments	Excess protein in the diet is an important risk factor, especially excess methionine from cheese. It leads to high homocysteine levels.
VLDL cholesterol is an important risk factor	1	Q3	LDL is more important than HDL but VLDL cholesterol is even more so.
Role of omega-3 in preventing CVD deaths	1	Q2	Omega 3 is anti-thrombotic and anti-arrhythmic. Synthetic supplements have been disappointing.

## Discussion

### Summary

Comments by participants (Table 5) seem to show a genuine interest in dietary factors that influence cardiovascular disease (CVD). Most GPs (97%) were well aware that diet is of crucial importance in the control of CVD (Tables 1 and 5). However, there was considerable confusion about certain aspects of this topic with 44% being “Not sure” about the role of omega-3 in recovery from cardiovascular events, and 33% being unsure what advice they should be giving on the safety of saturated fats in the diet (35% for female GPs). The lack of teaching on nutritional science in medical schools is illustrated by some responses from new recruits to general practice (Tables 3 and 4).

The uncertainty and doubts about the evidence regarding the heart and long chain omega-3 supplementation is illustrated by a response to the Question 2; “Omega-3 is key to recovery from a cardiac event” with the comment: “If it was, why was it taken off prescriptions?”, whereas others were impressed with the evidence for the anti-arrhythmic and anti-thrombotic activity of dietary long chain omega-3 fatty acids. Although controversial, there is good evidence for the anti-arrhythmic and anti-thrombotic properties of long chain omega-3 fatty acids [10–13]. However, there was an impression of general confusion rather than an informed consensus on this issue amongst these GPs.

While dietary *trans*-fatty acids were not mentioned in the questionnaire, six GPs commented on the importance of these fatty acids as a risk factor for CVD. There was some agreement on the role of dietary free sugars and saturated fats in CVD with 42.6% of GPs disagreeing with both the statement that dietary saturated fat is safe (Q4), and that dietary carbohydrates are neutral for CVD (Q7). Several comments highlighted the importance of the differences between free sugars (as a CVD risk factor) and complex carbohydrates, with the latter being generally viewed as being beneficial to cardiovascular health.

The split vote on the relative importance of LDL cholesterol and HDL cholesterol, probably reflects the current state of knowledge and confusion over the questionable clinical relevance of raised HDL cholesterol. However one participant suggested that the circulating level of VLDL (very low density lipoprotein) is more significant than either LDL or HDL. This may well be relevant to patients with cardiometabolic risk factors who are at risk of developing metabolic syndrome and type 2 diabetes mellitus and in whom serum LDL cholesterol is unremarkable. Although VLDL is not generally available for testing in primary care, estimation of 12 h fasting triacylglycerol (also termed triglycerides) is available and would be as reliable as VLDL as an indicator of cardiometabolic risk [19].

The overall outcome of this survey provides evidence of a body of professional doctors who have vested interest in diet and health. However, as several GPs indicated, their knowledge of the topic is inadequate and this may apply especially in the early stages of their career.

### Strengths and limitations

Strengths of this study include canvassing of grass roots GPs who are dealing with the issue of diet and CVD regularly. The main weakness of the study is the poor response rate to the initial e-mailed online survey monkey with only 89 (5.9% of targeted group) responding. However, this response rate is fairly typical for mailings to members of the RCGP. A recent election for places on the college council got an 8.4% response (personal communication from Professor Kamila Hawthorne). While these responders may represent a select group of GPs with a greater interest in nutrition, there was very little difference between the responders and the 19 non-responders. An exception was that more responders disagreed about the value of long chain omega-3 fatty acids for recovery from a cardiac event than non-responders (52.6% versus 33.7%, *p* = 0.076). Responders were twice as likely to opt for “Not sure” for this statement.

### Comparison with existing literature

Previous research in this area is limited to the teaching of nutritional science for medical students and associated academic departments [1–3], but tends to be in accord with the impression from this trial that teaching of nutritional science both in medical school and after medical school is inadequate.

### Implications for future research

To get an accurate assessment of the needs of GPs in relation to teaching on nutrition and health, a larger sample is needed. However, adequate coverage of this topic could be ensured by starting with focus groups to determine the best questions and format to use for any future research.

## Conclusions

GPs see dietary advice as part of their role, but do not consider themselves as experts. Despite the poor response rate, the value of this paper is that it can inform those designing diet/CVD related interventions for GP settings as it highlights information needs of GPs and maybe also practice nurses who are likely to deliver most of this care. The uncertainty about the interpretation of the nutrition science that underlies many of the diet-related issues that GPs experience could, perhaps, be rectified by raising the profile of nutrition on the curriculum in medical schools [1, 2].

## Additional files

Additional file 1: **Appendix 1**. Complete summary of trial data (raw study data used in SPSS analysis downloaded into an Excel file and edited to improve comprehension). (XLSX 20 kb)

Additional file 2: **Appendix 2**. Comparator data on Southern England GPs from other sources (data on age and gender balance from RCGP and government sources and statistical tests to show how they compared with our respondents). (DOCX 46 kb)

## Abbreviations

CVD: Cardiovascular disease
GP: General practitioner
HDL: High density lipoprotein
LDL: Low density lipoprotein
RCGP: Royal College of general Practitioners

## References

[1] Powell-Tuck J, Summerbell C, Garrow JS. Four years’ experience of an undergraduate medical nutrition course. J Roy Soc Med. 1997;90(2):67–72. 10.1177/014107689709000204 PMC1296140 9068433

[2] Long W, Neild P (2016). Nutrition training in UK medical undergraduate programmes – has the situation improved?. Gut.

[3] Womersley K, Ripullone K (2017). Medical schools should be prioritising nutrition and lifestyle education. BMJ.

[4] Dehghan M, Mente A, Zhang X, Swaminathan S, Li W, et al of the Prospective Urban Rural Epidemiology (PURE) study investigators. Associations of fats and carbohydrate intake with cardiovascular disease and mortality in 18 countries from five continents (PURE): a prospective cohort study. Lancet 2017;390: 2050–2062. 10.1016/S0140-6736(17)32252-3 28864332

[5] Ramsden CE, Domenichiello AF (2017). PUR study challenges the definition of a healthy diet: but key questions remain. Lancet.

[6] Wang DD, Hu FB (2017). Dietary fat and risk of cardiovascular disease: recent controversies and advances [review]. Annu Rev Nutr.

[7] Liu AG, Ford NA, Hu FB, Zelman KM, Mozaffarian D, Kris-Etherton PM (2017). A healthy approach to dietary fats: understanding the science and taking action to reduce consumer confusion. Nutr J.

[8] Cookson C. Studies confuse public debate on healthy eating, say experts. Financial Times Newspaper. https://www.ft.com/content/e9acd9b0-a5e0-11e3-b9ed-00144feab7de?segmentId=9b41d47b-8acb-fadb-7c70-37ee589b60ab. Accessed 26 Aug 18.

[9] NICE Guidelines. Myocardial infarction: cardiac rehabilitation and prevention of further cardiovascular disease. 2013. https://www.nice.org.uk/search?q=myocardial+infarction%3A+cardiac+rehabilitation. Accessed 26 Aug 2018. 31891465

[10] Leaf A, Albert CM, Josephson M, Steinhaus D, Kluger J, Kang JX, Cox B, Zhang H, Schoenfeld D, Fatty Acid Antiarrhythmia Trial Investigators. Prevention of fatal arrhythmias in high-risk subjects by fish oil n-3 fatty acid intake. Circulation. 2005;112(18):2762–8. 10.1161/CIRCULATIONAHA.105.549527 16267249

[11] Kromhout D, Geleijnse JM, de Goede J, Oude Griep LM, Mulder BJ, de Boer MJ, Deckers JW, Boersma E, Zock PL, Giltay EJ (2011). n-3 fatty acids, ventricular arrhythmia-related events, and fatal myocardial infarction in postmyocardial infarction patients with diabetes. Diabetes Care.

[12] Cao Y, Lu L, Liang J, Liu M, Li X, Sun R, Zheng Y, Zhang P (2015). Omega-3 fatty acids and primary and secondary prevention of cardiovascular disease [review]. Cell Biophys.

[13] Wang Q, Liang X, Wang L, Lu X, Huang J, Cao J, Li H, Gu D (2012). Effect of omega-3 fatty acids supplementation on endothelial function: a meta-analysis of randomized controlled trials [review]. Atherosclerosis.

[14] Khoueiry G, Abi Rafeh N, Sullivan E, Saiful F, Jaffery Z, Kenigsberg DN, Krishnan SC, Khanal S, Bekheit S, Kowalski M (2013). Do omega-3 polyunsaturated fatty acids reduce risk of sudden cardiac death and ventricular arrhythmias? A meta-analysis of randomized trials. Heart Lung.

[15] Billman GE (2013). The effects of omega-3 polyunsaturated fatty acids on cardiac rhythm: a critical reassessment [Review]. Pharmacol Ther.

[16] Higgins JPT, Capps NE, Riemersma RA, Ebrahim SBJ, Ness AR, Moore HJ, Worthington HV, Durrington PN, Hooper L, Thompson RL, Harrison RA, Summerbell CD (2006). Risks and benefits of omega 3 fats for mortality, cardiovascular disease, and cancer: systematic review. BMJ.

[17] Lentjes MAH, Keogh RH, Welch AA (2017). Longitudinal associations between marine omega-3 supplement users and coronary heart disease in a UK populationbased cohort. BMJ Open.

[18] Pealer LN (2001). The feasibility of a web-based surveillance system to college health risk behaviour. Health Ed Behav.

[19] Libby P (2015). Triglycerides on the rise: should we swap seats on the seesaw? [review]. Eur Heart J.

