# Supplementary material for: Responses to a GP survey: current controversies in diet and cardiovascular disease
Source: BMC Fam Pract. 2018 Aug 31;19:150. doi: 10.1186/s12875-018-0840-4 (PMC6119329; doi:10.1186/s12875-018-0840-4)
Supplement: Supplementary file 2 — Appendix 2. Comparator data on Southern England GPs from other sources (data on age and gender balance from RCGP and government sources and statistical tests to show how they compared with our respondents). (DOCX 46 kb) [file 12875_2018_840_MOESM2_ESM.docx]

**Current Controversies in diet and CVD**

**Appendix 2**

**Comparator data on Southern England GPs from other sources**

Mean values for gender balance for South England based on combined data from regional NHS workforce statistics (NHS Digital 2016. General and Personal Medical Services, England September 2015 - March 2016, Provisional Experimental statistics: [http://webarchive.nationalarchives.gov.uk/20180328140247/http://digital.nhs.uk/catalogue/PUB21772. Last accessed 13/5/18](http://webarchive.nationalarchives.gov.uk/20180328140247/http://digital.nhs.uk/catalogue/PUB21772.%20Last%20accessed%2013/5/18)) and data for South West Thames Faculty of the Royal College of General Practitioners (personal communication) were similar although there was a small bias in favour of female GPs responding to the questionnaire (68.5% of respondents compared with 59.3% of general sample). The NHS data also gives 30 years as the average age for entry into general practice so that estimated years in general practice were also similar( mean years as a GP: females 14.3 yr , males 19 Yr compared with respondents mean years as a GP: females 12 yr, males 17.6 yr).

**Regional South England GP sample from workforce survey for age & gender**

|  | Number of GPs | Mean ages | Std. deviation | Estimated years in GP (= approx -30 years) |
| --- | --- | --- | --- | --- |
| Male | 62 (40.8%) | 45.3 | 12.92 | 15.3 |
| Female | 90 (59.25%) | 40.2 | 9.49 | 10.2 |
| Total | 152 | 42.3 | 11.27 | 12.3 |

NB Sample of S England GPs from the NHS workforce survey who are all guaranteed either

full-time or part-time working GPs

**SWT Faculty analysis for age & gender based on members eligible for fellowship in 2013 (aged over 72 years excluded)**

|  | Number of GPs | Mean ages | Std. deviation | Estimated years in GP (= approx -30 years) |
| --- | --- | --- | --- | --- |
| Male | 292 (40.5%) | 52.6 | 8.5 | 22.6 |
| Female | 428 (59.4%) | 48.3 | 7.4 | 18.3 |
| Total | 720 | 50.06 | 8.15 | 20 |

These are GPs who as RCGP members might be suitable candidates for RCGP fellowship but an unknown proportion of those aged over 55 years old will have retired completely from general practice. They would, however, still be on the mailing list for SWT newsletters and therefore could have participated in the diet and CVD questionnaire.

**Combined data from regional workforce survey and SWT faculty**

|  | NHS workforce survey GP mean ages | SWT membership mean ages | Average of means | Estimated years in GP (= approx -30 years) | Averages of gender by %ge |
| --- | --- | --- | --- | --- | --- |
| Male | 45.3 | 52.6 | 49 | 19 | 40.7% |
| Female | 40.2 | 48.3 | 44.3 | 14.3 | 59.3% |
| Total | 42.3 | 50.06 | 46.2 | 16.2 |  |

**χ^2^ calculations to test significance of female GP bias in respondents compared with regional and SWT Faculty samples**

P = 0.12

P = 0.054

P = 0.54

Therefore, there was a statistical trend for the respondents to show a bias in favour of female GP responding to the questionnaire but this failed to reach statistical significance at p = 0.54.
